# The role of physiotherapy in the respiratory management of children with neuromuscular diseases: A South African perspective

**DOI:** 10.4102/sajp.v77i1.1527

**Published:** 2021-05-07

**Authors:** Anri Human, Lieselotte Corten, Brenda M. Morrow

**Affiliations:** 1 Department of Physiotherapy, Faculty of Healthcare Sciences, Sefako Makgatho Health Sciences University, Pretoria, South Africa; 2 Department of Health and Rehabilitation Sciences, Faculty of Health Sciences, University of Cape Town, Cape Town, South Africa; 3 Department of Physiotherapy, School of Health Sciences, University of Brighton, Eastbourne, United Kingdom; 4 Department of Paediatrics and Child Health, Red Cross War Memorial Children’s Hospital, University of Cape Town, Cape Town, South Africa

**Keywords:** respiratory management, neuromuscular diseases, chest physiotherapy, airway clearance techniques, children

## Abstract

**Background:**

Respiratory morbidity is common in children with neuromuscular diseases (NMD) owing to chronic hypoventilation and impaired cough. Optimal, cost-effective respiratory management requires implementation of clinical practice guidelines and a coordinated multidisciplinary team approach.

**Objectives:**

To explore South African physiotherapists’ knowledge, perception and implementation of respiratory clinical practice guidelines for non-ventilated children with NMD.

**Methods:**

An online survey was conducted amongst members of the South African Society of Physiotherapy’s Cardiopulmonary Rehabilitation (CPRG) and Paediatric special interest groups and purposive sampling of non-member South African physiotherapists with respiratory paediatrics expertise (*N*= 481).

**Results:**

Most respondents worked in private healthcare, with 1–10 years’ experience treating patients with NMD. For acute and chronic management, most participants recommended nebulisation and 24-h postural management for general respiratory care. Percussions, vibrations, positioning, adapted postural drainage, breathing exercises and manually assisted cough were favoured as airway clearance techniques. In addition, participants supported non-invasive ventilation, oscillatory devices and respiratory muscle training for chronic management.

**Conclusion:**

Respondents seemed aware of internationally-endorsed NMD clinical practice guidelines and recommendations, but traditional manual airway clearance techniques were favoured. This survey provided novel insight into the knowledge, perspectives and implementation of NMD clinical practice guidelines amongst South African physiotherapists.

**Clinical implications:**

There is an urgent need to increase the abilities of South African physiotherapists who manage children with NMD, as well as the establishment of specialised centres with the relevant equipment, ventilatory support and expertise in order to provide safe, cost-effective and individualised patient care.

## Introduction

Neuromuscular diseases (NMD) are a heterogeneous group of disorders that include pathology of the muscle (e.g. myopathies, muscular dystrophies), neuromuscular junction, peripheral nerves and motor neurons (e.g. anterior horn cell) (Yang & Finkel 2010). The types of NMD commonly seen in the paediatric population are muscular dystrophies (Duchenne and Becker muscular dystrophy [DMD and BMD]), spinal muscular atrophy (SMA) and congenital myopathies, with DMD presenting with the highest prevalence of one in every 3000–6000 live male births (Finder et al. 2004; Morrow et al. 2019; Yang & Finkel 2010). Spinal muscular atrophy is not as common as muscular dystrophies, with an estimated incidence of one in 10 000 live births for all types of SMA (Verhaart et al. 2017). Global prevalence for DMD and BMD has been estimated at 1.7–4.2 and 0.4–3.6 per 100 000, based on a synthesis of low risk of bias studies, whilst in some African countries such as Libya (DMD: 6/100 000) and Egypt (DMD: 7.7/100 000; BMD: 3.8/100 000), the prevalence is higher (Theadom et al. 2014). On the contrary, lower muscular dystrophy prevalence has been reported in South Africa (SA) (Theadom et al. 2014), with a minimum overall prevalence of one per 100 000 for DMD and one per 755 000 for BMD (*n* = 163) (Ballo, Viljoen & Beighton 1994). However, most NMD prevalence studies have been performed in small populations, primarily in European countries and prior to the use of genetic testing (Verhaart et al. 2017). Possible reasons for the observed difference in prevalence of NMD in SA compared to other countries, could be ascribed to the heterogeneity of the conditions, small number of South African prevalence studies available, differences in mutations between regions and certain ethnicities, decreased sensitivity of available tests and poor healthcare access for certain population groups (Ballo et al. 1994; Theadom et al. 2014; Verhaart et al. 2017).

Characterised by progressive muscle weakness, including cardiac and respiratory muscles, children with NMD often present with respiratory morbidity because of hypoventilation and an impaired cough (Chatwin et al. 2018; Farrero et al. 2013; Finkel et al. 2018). An ineffective cough leads to secretion retention and the potential for subsequent lower respiratory tract infection, airway obstruction, hypoventilation, dyspnea and sleep disturbances that can adversely affect health-related quality of life (Chatwin et al. 2018; Finkel et al. 2018; Morrow et al. 2019; Panitch 2006; Toussaint et al. 2018).

Even with new drug therapies changing the disease course and respiratory function over time in children with NMD, adequate symptomatic and preventative, pro-active respiratory management strategies are still recommended (Farrero et al. 2013; Landfeldt et al. 2015; Yang & Finkel 2010). Optimal, cost-effective and preventative respiratory management requires coordinated input from all the members of the multi-disciplinary team, including physiotherapists with experience in the management of children with NMD (Birnkrant et al. 2018; Chatwin et al. 2018; Farrero et al. 2013; Finkel et al. 2018). Furthermore, progressive respiratory muscle weakness in children with NMD may lead to postural deformities such as thoracic scoliosis, fibrosis of the chest wall muscles, decreased chest expansion and the development of progressive, restrictive pulmonary disease (Birnkrant et al. 2018; Farrero et al. 2013).

**FIGURE 1:**
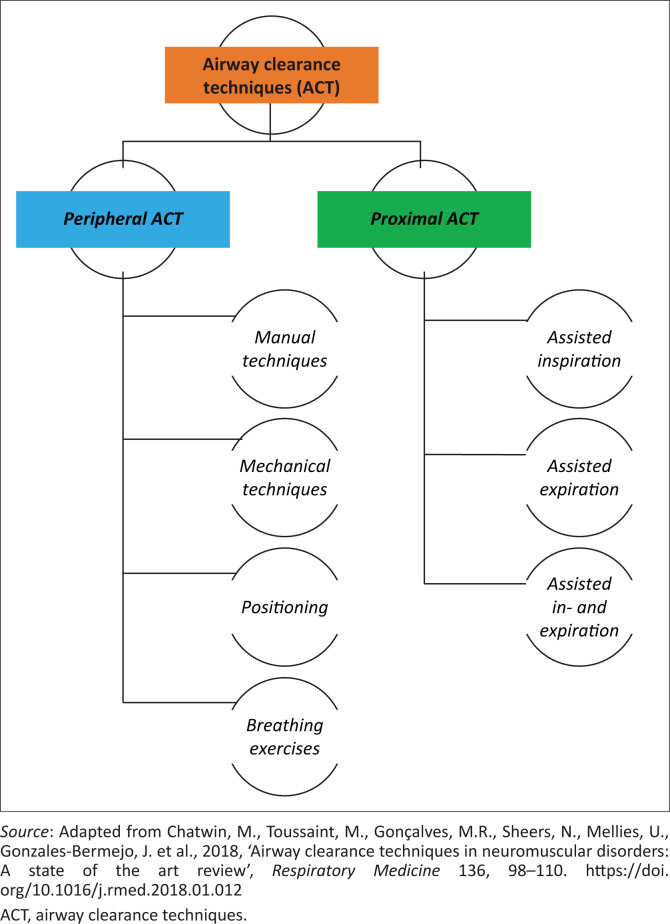
Peripheral and proximal airway clearance techniques, including cough augmentation.

Mechanical ventilation, oxygen supplementation as well as peripheral and proximal airway clearance techniques (ACT) can address the respiratory complications experienced by children with NMD (Chatwin et al. 2018; Finder 2010; McCool & Rosen 2006; Toussaint et al. 2018). Active respiratory physiotherapy is usually initiated once the child’s cough becomes ineffective, in order to assist with secretion mobilisation (peripheral ACT) and secretion clearance (proximal ACT) (Birnkrant et al. 2018; Chatwin et al. 2018; Finkel et al. 2018; Toussaint et al. 2018) (Figure 1).

Airway clearance techniques (Figure 1) aim to mobilise secretions from the peripheral to central airways (peripheral ACT), and/or assist with secretion clearance from the central airways (proximal ACT) (Chatwin et al. 2018; Hull et al. 2012; Toussaint et al. 2018; Wang et al. 2007). Peripheral ACT encompass manual techniques (percussions, shaking, vibrations, postural drainage [PD], positioning), breathing exercises (autogenic drainage, active cycle of breathing technique [ACBT]) and mechanical techniques (positive expiratory pressure [PEP] therapy, using oscillatory devices, high frequency chest wall compression or oscillation and intrapulmonary percussive ventilation) (Birnkrant et al. 2018; Finder 2010; Panitch 2006; Toussaint et al. 2018; Volsko 2013). Proximal ACT (cough augmentation) assists inspiration, expiration or both phases of the cough mechanism through a variety of manual and/or mechanical techniques such as manually assisted cough (MAC), lung volume recruitment (LVR) techniques and mechanical insufflation-exsufflation (MI-E) (Finder 2010; Finder et al. 2004; Toussaint et al. 2018). Airway clearance techniques are recommended in patients whose mucociliary escalator, mucus rheology, structural defects and/or poor cough mechanics, caused by muscle weakness, compromise their secretion mobilisation and expectoration (Farrero et al. 2013; McCool & Rosen 2006; Morrow et al. 2019; Toussaint et al. 2018).

Neuromuscular disease clinical practice guidelines, recommendations and updates for respiratory management have been published since 2004 and, if implemented, can minimise healthcare expenses whilst optimising patient outcome (Birnkrant et al. 2018; Chatwin et al. 2018; Finder et al. 2004; Finkel et al. 2018; Hull et al. 2012; Wang et al. 2007, 2010). In spite of the availability of clinical practice guidelines, management strategies and expertise vary between different settings and countries (Landfeldt et al. 2015; Wang et al. 2007, 2010). Presently, SA has a physiotherapist to patient ratio of 1:7511, which implies that the use of clinical practice guidelines could assist with high workload and improved management efficiency (Stander, Grimmer & Brink 2019). However, it is unknown whether South African physiotherapists (1) are aware of existing clinical practice guidelines and/or (2) have experience in working with children with NMD. Our study therefore aimed to explore the knowledge, perception and implementation of respiratory clinical practice guidelines in non-ventilated children with NMD (5–18 years) amongst physiotherapists in SA.

## Method

A quantitative, cross-sectional descriptive study within a non-probability purposive sampling frame was used. The target population consisted of all physiotherapists registered with the South African Society of Physiotherapy (SASP), with a self-identified special interest in cardiopulmonary rehabilitation (CPRG) and/or paediatrics, who were members of either one or both of these special interest groups.

South African physiotherapists with expertise in respiratory paediatrics, who were not members of the SASP special interest groups, were identified by the authors and invited to take part in the survey as their input could provide valuable information. Respondents without either academic, research or clinical expertise in the respiratory management of children with NMD were excluded from our study.

### Data collection tool and procedure

The self-constructed questionnaire was based on existing clinical practice guidelines and clinical expertise (Finder et al. 2004; Hull et al. 2012; Morrow et al. 2013; Wang et al. 2007, 2010). An expert panel of paediatric and/or pulmonology specialists from two tertiary institutions provided feedback and recommendations related to the content and structure of the questionnaire, thereafter the questionnaire was piloted (*n* = 5), prior to online distribution. The pilot study aimed to determine the approximate time for completion and whether questions were clear and unambiguous.

The final questionnaire consisted of four sections and participants were asked to indicate their support of specific respiratory management techniques (‘yes’, ‘no’, ‘unsure’, ‘patient dependent’ and open-text responses for additional comments).

#### Section A

Vocational background and clinical experience (in the field of NMD) of the participant.

#### Section B

Evidence-based respiratory management strategies were recommended for children with NMD in an acute setting (hospital), based on a case scenario:

Patient X has been diagnosed with a NMD and is older than five years of age and has recently become wheelchair-bound (non-ambulant). He has been admitted to hospital because of a respiratory infection and is presenting with a weak cough. He is haemodynamically stable, but oxygen saturation is 91%, and he is retaining secretions because of an ineffective cough.

The four subdivisions of Section B included questions relating to general respiratory care (including ventilatory support), peripheral ACT (secretion mobilisation), proximal ACT (cough augmentation) as well as other physiotherapy and general management strategies related to children with NMD during acute care.

#### Section C

Evidence-based respiratory management strategies were recommended for children with NMD in a chronic setting (home or school), based on a case scenario:

Patient Y has been diagnosed with a NMD and is older than five years of age. He recently became wheelchair-bound (non-ambulant) and attends follow-up visits at the neuro clinic every 3–6 months, depending on the need. Currently his vital capacity is < 80% predicted value for his age and he presents with a weak cough.

The four subdivisions of Section C enquired about ventilatory support, peripheral ACT and proximal ACT, LVR techniques (such as breath-stacking), breathing exercises, respiratory muscle training as well as other physiotherapy and general management strategies related to children with NMD during chronic management.

#### Section D

Respiratory management strategies used by South African physiotherapists in the acute and chronic settings, 6 months prior to the survey.

**FIGURE 2:**
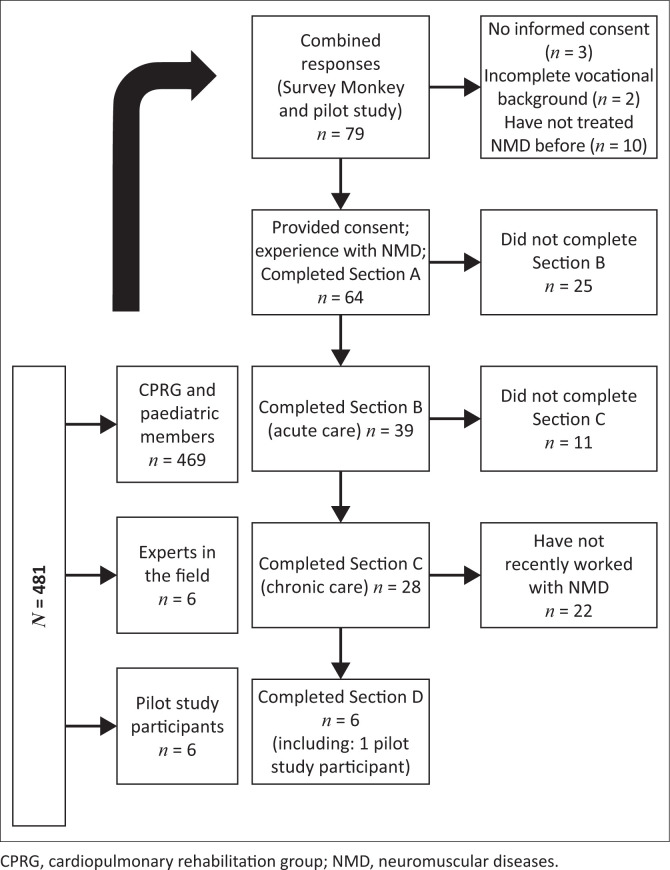
Flow chart of combined responses for pilot study participants and electronic responses.

The questionnaire (https://www.surveymonkey.com/r/8RF3F5J) was distributed electronically to 469 SASP members and six other physiotherapists with experience in NMD. Together with the pilot study participants, 481 eligible participants were requested to participate in the survey (Figure 2).

The survey system on Survey Monkey was opened for the participation of SASP members in June 2016 and reminder emails were sent approximately every 2 weeks, for a duration of 8 weeks.

### Data management and analysis

Data were entered into Excel spreadsheets, therafter the completed survey data (including pilot study responses) were exported to statistical programmes for a combined analysis STATA® (StataCorp, www.stata.com), Statistical Package for the Social Sciences (version 23) and STATISTICA® (Data Analysis Software System, Version 12, www.statsoft.com).

Depending on the normality of continuous data (tested using the Shapiro–Wilk *W*-test), the relevant parametric or non-parametric statistical tests were applied. Chi-square (χ^2^) tests and/or Yates χ^2^ tests (depending on the frequencies/responses per cell) were applied to determine the association between vocational background, physiotherapists’ years of experience and respiratory management techniques recommended. Statistical significance was accepted as *p* < 0.05.

Because of the distribution of responses and a variety of answers from participants for ‘repetitions’ and ‘frequency’ of treatment techniques, parametric tests could not be applied. Frequency tables, proportions and histograms or bar graphs illustrate descriptive statistics.

### Ethical considerations

The Institutional Review Board at the University of Cape Town provided ethical clearance (513/2015) and the SASP Executive Committee (president), as well as the chairpersons of CPRG and Paediatric Special Interest Group granted permission to distribute the questionnaire to their members. Informed consent was obtained from participants by including study information and a confirming consent statement on the opening page of the Survey Monkey questionnaire.

## Results

A flow chart of survey responses is presented in Figure 2. The link to the survey was distributed online to 469 SASP members and, through purposive sampling, to six experts and to six eligible pilot study participants (*N* = 481). Minimal changes were made to the questionnaire following the pilot study and therefore these responses (*n* = 5) were included in the final analysis.

The total number of recruited participants consisted of 74 physiotherapists who responded to the online survey, as well as five out of the six pilot study participants with a response rate of 16.4% (79/481).

Three of the initial respondents did not provide consent; two provided incomplete vocational information (Section A) and 10 reported that they had not previously worked with children with NMD or had no experience in the field. These 15 participants were therefore excluded from the original 79 responses. The final sample of **64** responses was included in the final analysis, which constitutes a response rate of 13.3% (64/481). The number of responses, however, diminished with every section of the questionnaire (Figure 2).

### Section A: Vocational background and experience of participants (*n* = 64)

Questions on vocational background (area of work) allowed for more than one option to be chosen. The majority of the 64 participants worked in the private sector which included out-patients and hospital wards (general and specialised) (58%; *n* = 37) as compared to approximately 42% (*n* = 27) working in the public healthcare sector including hospitals (general and specialised wards), special schools, rehabilitation centres or clinics and universities.

Most participants (42%; *n* = 27) reported 1–5 years of experience working with NMD, and two-third of respondents (66%; *n* = 43) indicated between 1 and 10 years of experience in this field.

No statistically significant association was found between years of experience in NMD and clinical practice trends such as ventilatory support, oxygen supplementation, ACT and respiratory muscle training. The use of LVR (breath-stacking) during chronic management of children with NMD, however, showed a significant association with the place of work (public vs. private healthcare sector) (Yates χ^2^, [1; *n* = 28] = 4.74, *p* = 0.03). Participants working in public healthcare were more in favour of the use of breath-stacking during chronic management (*n* = 8/12) than those from the private sector (*n* = 3/16).

### Section B: Acute management for non-ventilated children and adolescents with neuromuscular diseases (*n* = 39–44)

In this section of the questionnaire, participants were asked about recommended evidence-based respiratory management strategies specifically related to general respiratory care as well as peripheral and proximal ACT during acute management of children with NMD.

**FIGURE 3:**
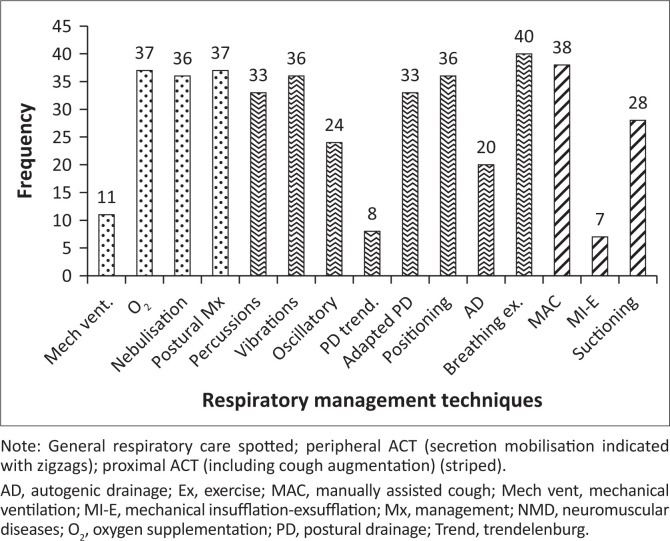
Acute respiratory management in children and adolescents with neuromuscular diseases (*n* = 39–44).

A varying number of participants (*n* = 39–44) completed Section B on acute management (Figure 3).

Under general respiratory management (indicated in Spotted, Figure 3), supplemental oxygen delivery (40%, 2 L – 5 L), nebulisation (saline or saline and bronchodilators or mucolytics and 24-h postural management (supported sitting; adapted side-lying and semi-Fowler’s) were mostly recommended. For mechanical ventilation, most of the participants indicated that they would use non-invasive ventilation (NIV) as a mechanical ventilation option.

Peripheral ACT (indicated in Zigzag, Figure 3) mostly recommended were percussions, vibrations, adapted PD (no Trendelenburg positioning), using oscillatory devices and breathing exercises (e.g. ACBT and/or deep breathing exercises).

Manually assisted cough, with a combination of thoraco-abdominal compression, was mostly indicated as proximal ACT, whilst suctioning was also well supported (Striped, Figure 3; Table 1). As a safety precaution, the majority of participants indicated that the cough-assisted technique used should either be changed or stopped when the patient becomes tired.

**TABLE 1:** Summary of suggested treatment duration and frequencies for peripheral and proximal airway clearance techniques (acute care).

Treatment	Duration per treatment (min)	Proportion	%	Frequency (per day)	Proportion	%
Percussions	10 or 15	9/31	29	2	23/31	74
Vibrations	5 or 10	12/33	36	2	24/33	73
Postural drainage (Trendelenburg)	1–20	N/A		2	6/8	75
Adapted postural drainage (No Trendelenburg positioning)	10 or 20	7/30	23	2	18/28	64
Positioning	10 or 20 or 30	8/32	25	2	15/29	52
Autogenic drainage (Including assisted autogenic drainage)	5 or 10	6/19	32	2	13/18	72
Breathing exercises: Active cycle of breathing (including modified ACBT) (18/36) Deep breathing exercises (alone or in combination) (9/36)	5–10	15/35	43	2	18/32	56
Manually assisted cough	Frequency per treatment: 3–5 or 5 or 5–10 coughs per treatment	6/32	19	Patient dependent	27/38	71
Suctioning	Frequency per treatment: 1 (once)	8/22	36	2	10/22	46

ACBT, active cycle of breathing technique.

Table 1 contains the treatment duration and frequency of peripheral and proximal ACT as recommended by the majority of participants. There was, however, a varying number of responses for the duration and frequency of ACT, therefore proportions were also indicated.

Only a minority of participants (*n* = 7) recommended alternative cough augmentation techniques such as MI-E, and 22 participants indicated that they were unsure of the use of these devices.

Additional recommendations for ACT during acute management provided by survey participants as open-text responses included mobilisation (change of position, thoracic or upper limb mobility), breathing exercises (huffing, breath-stacking, ACBT, PEP therapy using blow bottle or windmill blowing and respiratory muscle training) as well as caregiver education and/or support.

### Section C: Chronic management for non-ventilated children and adolescents with neuromuscular diseases (*n* = 28)

**FIGURE 4:**
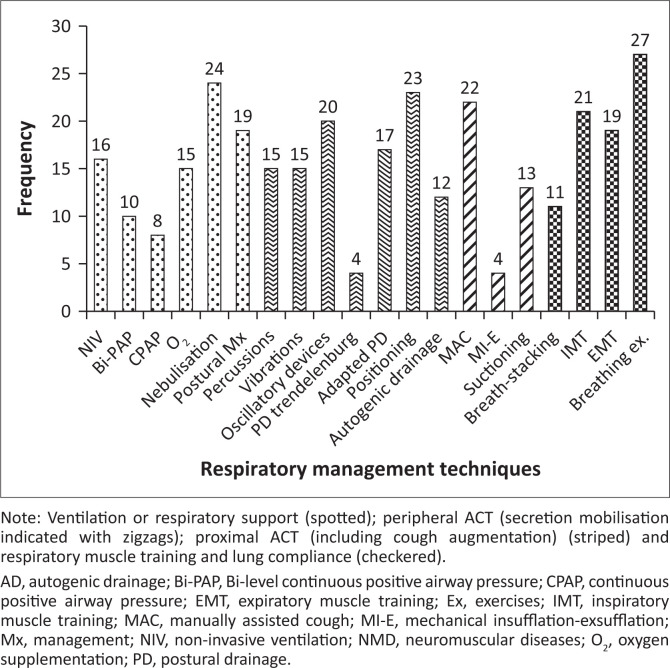
Chronic management in children and adolescents with neuromuscular diseases (*n* = 28).

The responses (*n* = 28) for chronic management are depicted in Figure 4.

Under ventilatory support during chronic management (indicated in Spotted, Figure 4), many respondents recommended NIV (Bi-level Positive Airway Pressure [Bi-PAP] or Continuous Positive Airway Pressure [CPAP]), 24-h postural management (high or supported sitting), oxygen supplementation and nebulisation (saline or saline combined with mucolytics).

The majority of the participants recommended oscillatory devices, positioning and adaptive PD for peripheral ACT (Zigzag, Figure 4) to enhance secretion mobilisation.

Similar to acute care, MAC was strongly recommended for cough augmentation as part of proximal ACT, favouring a combined thoraco-abdominal technique. Most of the participants were unsure of the use of cough assist devices (MI-E) (Striped, Figure 4). Slighty fewer than 50% (13/27) of respondents supported the use of suctioning to assist with airway clearance during chronic management.

Regarding the use of lung compliance (LVR), breathing exercises and respiratory muscle training (Checkered, Figure 4), half of the participants were unsure about the use of breath-stacking (independent or assisted or combination technique), manual inflation or glossopharyngeal breathing (GPB) during chronic management. The majority of participants, however, recommended the use of breathing exercises (ACBT, bubble blowing), and more than two-thirds of the participants suggested inspiratory and/or expiratory muscle training (IMT or EMT).

Treatment duration and frequency of peripheral and proximal ACT as well as respiratory muscle training during chronic care of children with NMD, as recommended by the majority of participants, are depicted in Table 2. Because of varying number of responses for the duration and frequency of ACT, proportions were also indicated.

**TABLE 2:** Summary of suggested treatment duration and frequencies for respiratory muscle training, lung compliance and airway clearance techniques (chronic care).

Treatment	Repetitions per day (min)	Proportion	%	Frequency per day	Proportion	%
Percussions	Time per treatment: 10	5/12	42	2	8/12	67
Vibrations	Time per treatment: 5	4/12	33	2	5/12	42
Oscillatory devices	Frequency per treatment: 10 times	4/18	22	2	7/18	39
Adapted postural drainage (No Trendelenburg positioning)	Time per treatment: 10 20	4/14	29	2	6/14	43
Positioning	Time per treatment: Patient dependent	4/19	21	2	5/17	29
Autogenic drainage	Time per treatment: Patient dependent	3/10	30	2	5/10	50
Manually assisted cough	Coughs per treatment: Patient dependent; as needed	9/17	53	Patient dependent	19/26	73
Suctioning	Frequency per treatment: As required; as needed	6/12	50	As required; as needed; patient dependent	6/9	67
Inspiratory muscle training (One participant was unsure)	5 10 Patient dependent	2/19 3/19 3/19	11 16 16	2 Patient dependent	6/19 4/19	32 21
Expiratory muscle training (Five participants were unsure)	5 10 Patient dependent	2/16 4/16 3/16	13 25 19	2 Patient dependent	5/16 3/16	31 19
Breathing exercises	Time per treatment: 5	5/22	23	2	11/22	50

Additional comments under the open-text responses related to ACT included the use of hydrotherapy for improved respiratory function, breathing exercises (especially ACBT), percussions, the use of nebulised normal saline for secretion mobilisation, suctioning only if needed and the use of MI-E using both manual and automatic settings. Furthermore, general recommendations for chronic management in NMD included pharmacological intervention, nutrition and hygiene as well as including exercise or games or play therapy in the patients’ home programme to improve their respiratory function.

A summary of the favoured respiratory management treatment strategies, for both acute and chronic management, is provided in Table 3.

**TABLE 3:** Summary of preferred techniques in acute and chronic management of children with neuromuscular diseases.

Management	Acute (*n* = 39–44)	Chronic (*n* = 28)
General respiratory care or respiratory support	Non-invasive ventilation Nebulisation (normal saline) Supplemental O_2_ 24-h postural management	Non-invasive ventilation Supplemental O_2_ 24-h postural management
Secretion mobilisation	Percussions Vibrations Adapted postural drainage (No Trendelenburg) Positioning Oscillatory devices Breathing exercises	Nebulisation Adapted postural drainage (No Trendelenburg) Positioning Oscillatory devices
Airway clearance and cough augmentation or assistance	Manually assisted cough† (Thoracic and abdominal compressions) Suctioning	Manually assisted cough† (Thoracic and abdominal compressions)
Lung compliance exercises and respiratory muscle training	Not included as part of acute management	Breathing exercises^‡^ Inspiratory and expiratory muscle training

O_2_, oxygen supplementation.

† , The majority of the participants were either not aware of MI-E as an alternative cough augmentation option or were aware of the device, but had never used it before.

‡ , 50% of participants were unsure about the use of LVR such as breath-stacking or GPB.

### Section D: Respiratory management strategies used by South African physiotherapists 6 months prior to the survey (*n* = 6)

Six (10%) participants responded to this section, as they treated children with NMD on a regular basis and had used respiratory management strategies in this patient cohort within 6 months prior to the survey.

All respondents (*n* = 6) used adapted PD, positioning and breathing exercises during both acute and chronic management of children with NMD. In addition, all participants (*n* = 6) used vibrations during acute care and most physiotherapists (*n* = 5) used percussions for acute and chronic management and respiratory muscle training during chronic care.

Mechanical insufflation-exsufflation and LVR or lung compliance techniques such as breath-stacking and GPB were not used for either acute or chronic management.

## Discussion

### Respondent background and South African context

All practicing South African physiotherapists are obligated to register with the Health Professionals Council of South Africa (HPCSA), whilst membership of other professional bodies such as the SASP is voluntary and requires additional annual membership fees. Of the South African physiotherapists registered with the HPCSA, approximately 54% are members of the SASP, most of whom work in private healthcare (Fourie 2019; Stander et al. 2019; World Physio n.d. [https://world.physio/membership/south-africa]). Selection bias might, therefore, be present, with inadequate representation of physiotherapists working in the South African public healthcare sector. For this reason, future surveys should be conducted via more inclusive platforms such as the HPCSA.

Most respondents (66%) indicated 1–10 years of NMD experience, but we were unable to show an association between years of experience and clinical practice trends. There was one statistically significant association (*p* = 0.03) between place of work and recommended respiratory management strategies. Participants working in public healthcare seemed more inclined to use breath-stacking techniques (LVR) for chronic management in children with NMD than those in the private healthcare sector. The reasons for the difference between the healthcare sectors are unknown and should be interpreted with caution because of the small sample size, and therefore, this warrants further research. A recent South African based study, however, indicated that the barriers and facilitators to the implementation of clinical practice guidelines amongst physiotherapists seem to be similar, regardless of geographical location or work context (private or public healthcare) (Stander et al. 2019).

The low response rate (13.3%) limits the generalisability of the survey results, however, the response rate was similar to another survey conducted amongst SASP special interest groups and higher than other online surveys conducted amongst physiotherapists (Clenzos, Naidoo & Parker 2013; Silva, Costa & Costa 2015; Struyf et al. 2012). Respondent bias is also a limitation of our study, because physiotherapists who were more knowledgeable about NMD may have been more likely to complete the survey. Of concern was that the attrition rate in our survey increased from Section B (acute care) to Section D (South African management strategies), leading to a varying number of responses per section. Possible reasons could include the nature and/or length of the questionnaire or limited expertise regarding either acute or chronic management of children with NMD (Stander et al. 2019; Toussaint et al. 2018). The low number of responses provided for Section D (*n* = 6) might not be a true reflection of the physiotherapy management strategies used in SA, but could allude to the fact that few physiotherapists are involved in the management of children with NMD.

### Acute and chronic management of non-ventilated children with neuromuscular diseases

The case scenarios both described non-ambulant children presenting with decreased pulmonary function and a poor cough. Similar to clinical recommendations for non-ambulant children with NMD, presenting with associated restrictive lung disease and pulmonary function regression, most survey participants supported NIV during acute (14/44) and especially chronic management (16/27) (Birnkrant et al. 2018; Finkel et al. 2018; Hull et al. 2012). Respondents recommended using Bi-PAP, rather than CPAP, which aligns with clinical recommendations. The use of CPAP alone does not necessarily decrease the ventilatory load, may fatigue the patient and could interfere with weaning, whilst Bi-PAP is likely to be more effective in addressing hypoventilation and may be more comfortable (Finder et al. 2004; Finkel et al. 2018; Wang et al. 2007).

Participants also recommended the use of nebulisation, 24-h postural management and oxygen supplementation during acute and chronic management. Clinical practice guidelines on nebulisation and postural management strategies in children with NMD are limited and are, therefore, usually based on clinical reasoning and patient presentation, as indicated by many participants. Survey participants also seemed to be aware that the chronic use of mycolytics such as hypertonic saline in children with NMD is not advisable, but that inhaled bronchodilators may be considered in children presenting with asthma or bronchial hyperresponsiveness (bronchospasm) (Finkel et al. 2018; Piper & Moran 2006; Wang et al. 2007).

Positioning of patients such as side-lying and a variety of sitting positions during acute and chronic management was strongly supported by participants, most likely for the benefit of regional lung ventilation redistribution, resolving unilateral and/or isolated lung infiltration, improved secretion mobilisation and delay of secondary complications (Lupton-Smith et al. 2014; Toussaint et al. 2018). This aligns with the physiological principle that functional residual capacity is influenced by positioning and improves progressively from supine to standing (Lumb & Thomas 2020). In addition, lung compliance can be improved and airway resistance decreased by changing a patient’s position from supine (Wahba 1991). Postural management may enhance regional lung ventilation and/or secretion mobilisation, especially during respiratory infections, but can pose a challenge because of secondary complications in patients with NMD such as scoliosis, contractures and osteoporosis, particularly once patients lose ambulation (Panitch 2006; Toussaint et al. 2018).

The acute case scenario indicated desaturation (SpO_2_ < 95%), which could warrant the use of supplemental oxygen. However, hypoxaemia in children with NMD is usually caused by hypoventilation, mucus plugging, atelectasis and/or a respiratory tract infection. Supplemental oxygen should then rather be combined with ventilatory support such as NIV and cough augmentation (proximal ACT) in order to address the underlying cause(s) of the desaturation (Birnkrant et al. 2018; Finkel et al. 2018). The majority of respondents supported the use of oxygen supplementation during acute care, whilst only a few specified that the dosage and use should be based on the patient’s needs. Most participants correctly indicated the use of NIV and ACT (peripheral and proximal) once a patient becomes non-ambulant, presents with an acute infection, saturation of < 95% on room air and presents with secretion retention because of poor cough ability, as outlined in the case scenarios (Birnkrant et al. 2018; Chatwin et al. 2018; Farrero et al. 2013; Finkel et al. 2018).

Secretion management with ACT is advocated in order to maintain health, improve health-related quality of life and minimise respiratory complications, which may explain why these techniques were well supported by respondents (Birnkrant et al. 2018; Finder 2010; Finkel et al. 2018). Survey participants recommended the use of peripheral ACT twice per day or based on the patient’s needs, with treatment time varying from 5 min to 20 min, similar to other studies on patients presenting with secretion retention (Balachandran, Shivbalan & Thangavelu 2005; Piper & Moran 2006). Peripheral ACT such as percussions, vibrations and PD aim to enhance secretion mobilisation, but may be associated with adverse effects such as desaturation, bronchospasm, increase in gastro-oesophageal reflux, blood pressure and rib injuries (Balachandran et al. 2005; Panitch 2006; Rous et al. 2014). Furthermore, the efficacy of techniques such as percussion and vibrations, combined with PD, may be limited in children with NMD because of the presence of scoliosis, contractures and/or osteoporotic ribs (Panitch 2006; Toussaint et al. 2018). The majority of participants correctly indicated avoiding the use of head down PD (Trendelenburg) as this technique could cause harm in the paediatric population (Morrow 2019), therefore adapted PD should rather be used during acute and chronic management.

Utilising oscillatory PEP devices for secretion mobilisation was also better supported by respondents during chronic management than during acute management. All the South African physiotherapists who recently worked with children with NMD (*n* = 6) reported a preference for more conventional peripheral ACT such as adapted PD, positioning and breathing exercises during acute and chronic management. The majority also used percussions, vibrations and EMT. Although these ACT might have clinical merit and are supported by clinical practice guidelines, they do have limitations and precautionary measures should be considered. Breathing exercises, incentive spirometry and PEP might not be as effective in patients with NMD because of their underlying respiratory muscle weakness, unless concomitant ventilatory support is also provided (Birnkrant et al. 2018; Chatwin et al. 2018; Finder 2010; Toussaint et al. 2018). Bubble blowing (PEP) and ACBT were strongly supported for chronic management by nearly all survey participants. The disadvantage of these breathing exercises is that they are effort and expiratory flow dependent, therefore diminishing their efficacy in non-ambulant patients with progressed disease, pulmonary function decline and severe respiratory muscle weakness (Finder 2010; McCool & Rosen 2006; Toussaint et al. 2018). In addition, the majority of survey participants supported the use of respiratory muscle training during chronic management, despite limited evidence. Respiratory muscle training is not currently recommended as a standard therapy in children with NMD, but these devices are available and therefore larger, randomised controlled trials are recommended to determine their safety, feasibility and efficacy within a South African context (Finder et al. 2004; Human et al. 2017).

Survey participants correctly indicated the need for cough assistance based on the patient presentation (decreased vital capacity, desaturation and poor cough), which aligns with clinical practice guidelines (Birnkrant et al. 2018; Finkel et al. 2018; Hull et al. 2012). Cough effort can be enhanced with manual and/or mechanical techniques by assisting inspiration (e.g. breath-stacking), expiration (MAC) or both (e.g. with MI-E) (Chatwin et al. 2018; Finder 2010; Finder et al. 2004; McCool & Rosen 2006; Panitch 2006). Manual cough assistance was very well supported by survey participants, with most recommending a combination of thoraco-abdominal thrusts or compressions, for both acute and chronic management of children with NMD, as recommended by clinical practice guidelines (Bianchi & Baiardi 2014; Farrero et al. 2013; Finder 2010; Toussaint et al. 2018). Unfortunately, MAC can be labour-intensive, mostly requires assistance, difficult to perform by non-professionals and requires a coordinated effort by the caregiver and patient. Furthermore, MAC may be poorly tolerated and at times even ineffective, especially in patients with post-abdominal surgery and those with scoliosis, restrictive chest walls, osteoporosis, obesity or intra-abdominal catheters (Chatwin et al. 2018; Finder 2010; McCool & Rosen 2006; Panitch 2006). Nonetheless, MAC is a cost-effective cough augmentation technique recommended for patients with NMD from an early age and can easily be used within the South African context, as was evident from the survey responses (Chatwin et al. 2018; Finder et al. 2004).

On the contrary, MI-E devices are recommended and used in many developed, high-income countries as an alternative cough augmentation method to curb respiratory deterioration (Chatwin et al. 2018; Farrero et al. 2013; Finder 2010; Finkel et al. 2018; Panitch 2006). Mechanical cough assistance can be especially useful in patients with severe respiratory muscle weakness, frequent respiratory infections, decreased oral or bulbar control and/or those unable to cooperate with manual insufflation and MAC, or if these techniques have been shown to be unsuccesful (Chatwin et al. 2018; Farrero et al. 2013; Hull et al. 2012; Toussaint et al. 2018). Challenges with MI-E include availability of devices and cost implications, heterogeneity in application, limited or low-level evidence and safety concerns, especially in the paediatric population (Castrillo et al. 2019; Chatwin et al. 2018; Morrow et al. 2013; Toussaint et al. 2018). Although the use of MI-E is recommended in patients with NMD, these cough assist devices and the clinical expertise to use them might not always be available in middle- and low-income countries such as SA (Birnkrant et al. 2018; Chatwin et al. 2018; Finder et al. 2004; Finkel et al. 2018; Toussaint et al. 2018). This survey highlighted the limited use and possible lack of knowledge regarding mechanical cough assist devices, as none of the physiotherapists used MI-E in the 6 months prior to the survey. Only a few participants recommended the use of MI-E for the acute (*n* = 7) and chronic (*n* = 4) scenario, respectively, despite NMD clinical practice guideline recommendations and the progressed respiratory disease of the patients outlined in the case scenarios. Only two participants responded to questions related to MI-E settings for acute and chronic management, and therefore results may not be a true reflection of knowledge or opinion of most physiotherapists in SA.

Another cough augmentation technique, LVR or breath-stacking, can be performed manually (e.g. with an ambubag) or mechanically (with a ventilator or cough assist device) and is recommended in patients with NMD. The benefits of LVR include secretion mobilisation, improved ventilation, cough ability and maintaining lung and chest compliance with the aim of decreasing pulmonary function regression and preventing respiratory morbidity (Castrillo et al. 2019; Chatwin et al. 2018; Farrero et al. 2013; Hull et al. 2012; Toussaint et al. 2018). Combining insufflation and exsufflation seems to be well-tolerated and more efficient than any of the techniques as a stand-alone, especially once patients become non-ambulant and develop scoliosis (Hull et al. 2012; Toussaint et al. 2018).

Besides these advantages, half the survey participants were unsure about the use of LVR during chronic management and only a minority supported the use of cough augmentation techniques such as breath-stacking and GPB (Castrillo et al. 2019; Farrero et al. 2013; McCool & Rosen 2006; Toussaint et al. 2018). Various factors could be responsible for the limited use of alternative cough augmentation techniques, including resource constraints, because MI-E devices have to be imported and are therefore very costly, and also because of possible limited exposure and/or knowledge of physiotherapists in using LVR and MI-E. In addition, environmental factors such as lack of electricity, especially in the rural areas of SA, might also influence the use of devices and, therefore, manual insufflation and MAC might be more cost-effective alternatives to consider (Finder 2010).

The chronic case scenario depicted a non-ambulant patient with progressed disease and a poor cough effort. Possibly because of the clinical presentation, survey participants could have assumed that the patient would present with severe respiratory muscle weakness and possibly decreased oral control. Not all patients are able to perform GPB or spontaneous breath-stacking as these techniques require good oral control and coordination (Farrero et al. 2013; Toussaint et al. 2018). Aligned with clinical practice guidelines, breath-stacking was, however, used by the physiotherapists who treated children with NMD 6 months prior to the survey (4/6 for acute and 3/6 for chronic). Availability of ambu-bags, ventilators and cough augmentation devices, variation in implementation and experience or training might also affect the use of LVR techniques (Castrillo et al. 2019; Chatwin et al. 2018).

## Conclusion

In SA, physiotherapists as first-line practitioners play a primary role in the multidisciplinary team responsible for evidence-based respiratory management of children with NMD in order to prevent, delay and/or manage respiratory complications (Birnkrant et al. 2018; Chatwin et al. 2018; Finkel et al. 2018; Landfeldt et al. 2015). Respondents were generally aware of international NMD clinical practice guidelines and recommendations, but traditional, manual ACT were favoured (Finkel et al. 2018; Landfeldt et al. 2015; McCool & Rosen 2006; Stander et al. 2019). The implementation of internationally endorsed clinical practice guidelines might be influenced by the heterogeneity of NMD, lack of human and/or financial resources, knowledge and/or skill and support from management, limited exposure, technological advances, time-constraints and institutional or environmental barriers (Chatwin et al. 2018; Stander et al. 2019; Toussaint et al. 2018).

Specialist care for NMD in SA is limited and there is an urgent need to increase the abilities of South African physiotherapists as well as the establishment of specialised centres with the relevant equipment, ventilatory support and expertise (Birnkrant et al. 2018; Farrero et al. 2013; Landfeldt et al. 2015; Stander et al. 2019). Raising awareness and providing access to clinical practice guidelines, equipment and training (e.g. through workshops or courses) might increase knowledge and facilitate the implementation of NMD practice guidelines amongst South African physiotherapists (Stander et al. 2019; CPRG mission statement [SASP]). Although the use of ACT may be of clinical benefit in children with NMD, evidence for efficacy, long-term outcome and safety in this population is limited (Birnkrant et al. 2018; Chatwin et al. 2018; Finkel et al. 2018; Volsko 2013). Multi-centre randomised controlled trials and the development of context-specific clinical practice guidelines are, therefore, recommended to establish safe, effective and feasible respiratory management strategies for children with NMD in SA (Birnkrant et al. 2018; Stander et al. 2019; Volsko 2013).

This survey provided novel insight into the knowledge, perspectives and implementation of NMD clinical practice guidelines amongst South African physiotherapists. Results from this survey can also inform healthcare managers and policymakers, especially in light of the proposed changes to the South African healthcare system, as well as planning for future clinical trials.
