# Erratum: The role of physiotherapy in the respiratory management of children with neuromuscular diseases: A South African perspective

**DOI:** 10.4102/sajp.v77i1.1616

**Published:** 2021-11-22

**Authors:** Anri Human, Lieselotte Corten, Brenda M. Morrow

**Affiliations:** 1 Department of Physiotherapy, Faculty of Healthcare Sciences, Sefako Makgatho Health Sciences University, Pretoria, South Africa; 2 Department of Health and Rehabilitation Sciences, Faculty of Health Sciences, University of Cape Town, Cape Town, South Africa; 3 Department of Physiotherapy, School of Health Sciences, University of Brighton, Eastbourne, United Kingdom; 4 Department of Paediatrics and Child Health, Red Cross War Memorial Children’s Hospital, University of Cape Town, Cape Town, South Africa

In the version of the article initially published, Human, A., Corten, L. & Morrow, B.M., 2021, ‘The role of physiotherapy in the respiratory management of children with neuromuscular diseases: A South African perspective’, *South African Journal of Physiotherapy* 77(1), a1527. https://doi.org/10.4102/sajp.v77i1.1527, the publication year was given incorrectly. The correct publication date should be 07 May 2021 instead of 07 May 2020 in the ‘Dates’ section.

This correction does not alter the study’s findings of significance or overall interpretation of the study’s results. The publisher apologises for any inconvenience caused.

